# A biophysical observation model for field potentials of networks of leaky integrate-and-fire neurons

**DOI:** 10.3389/fncom.2012.00100

**Published:** 2013-01-04

**Authors:** Peter beim Graben, Serafim Rodrigues

**Affiliations:** ^1^Bernstein Center for Computational Neuroscience BerlinBerlin, Germany; ^2^Department of German Language and Linguistics, Humboldt-Universität zu BerlinBerlin, Germany; ^3^Centre for Robotics and Neural Systems, School of Computing and Mathematics, University of PlymouthPlymouth, UK

**Keywords:** biophysics, neural networks, leaky integrate-and-fire neuron, current dipoles, extracellular medium, field potentials

## Abstract

We present a biophysical approach for the coupling of neural network activity as resulting from proper dipole currents of cortical pyramidal neurons to the electric field in extracellular fluid. Starting from a reduced three-compartment model of a single pyramidal neuron, we derive an observation model for dendritic dipole currents in extracellular space and thereby for the dendritic field potential (DFP) that contributes to the local field potential (LFP) of a neural population. This work aligns and satisfies the widespread dipole assumption that is motivated by the “open-field” configuration of the DFP around cortical pyramidal cells. Our reduced three-compartment scheme allows to derive networks of leaky integrate-and-fire (LIF) models, which facilitates comparison with existing neural network and observation models. In particular, by means of numerical simulations we compare our approach with an *ad hoc* model by Mazzoni et al. (2008), and conclude that our biophysically motivated approach yields substantial improvement.

## 1. Introduction

**Figure 1:**
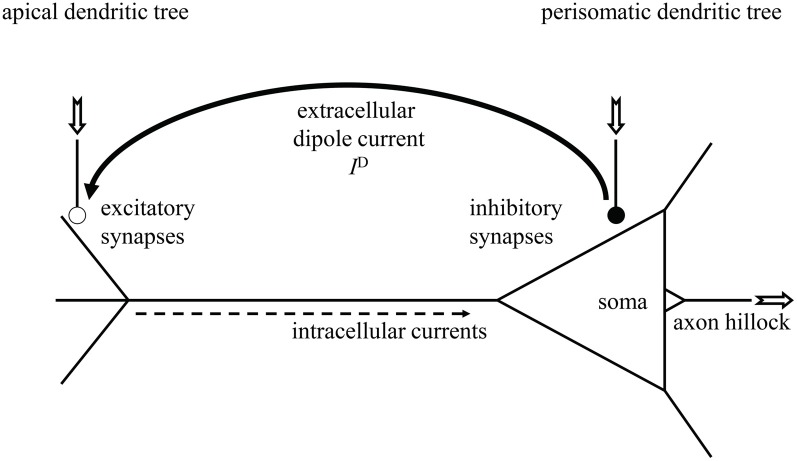
**Sketch of a cortical pyramidal neuron with extracellular current dipole between spatially separated excitatory (open bullet) and inhibitory synapses (filled bullet).** Neural in- and outputs are indicated by the jagged arrows. Dendritic current *I*^D^ causes dendritic field potential (DFP).

Since Hans Berger's 1924 discovery of the human *electroencephalogram (EEG)* (Berger, 1929), neuroscientists achieved much progress in clarifying its neural generators (Creutzfeldt et al., 1966a,b; Nunez and Srinivasan, 2006; Schomer and Lopes da Silva, 2011). These are the cortical pyramidal neurons, as sketched in Figure 1, that possess a long dendritic trunk separating mainly excitatory synapses at the apical dendritic tree from mainly inhibitory synapses at the soma and at the perisomatic basal dendritic tree (Creutzfeldt et al., 1966a; Spruston, 2008). In addition, they exhibit an axial symmetry and are aligned in parallel to each other, perpendicular to the cortex' surface, thus forming a palisade of cell bodies and dendritic trunks. When both kinds of synapses are simultaneously active, inhibitory synapses generate current sources and excitatory synapses current sinks in extracellular space, hence causing the pyramidal cell to behave as a microscopic dipole surrounded by its characteristic electrical field, the *dendritic field potential (DFP)*. The densely packed pyramidal cells form then a dipole layer whose superimposed currents give rise to the *local field potential (LFP)* of neural masses and eventually to the EEG (Nunez and Srinivasan, 2006; Lindén et al., 2010; Lindén et al., 2011; Schomer and Lopes da Silva, 2011).

Despite of the progress from experimental neuroscience, theoretically understanding the coupling of complex neural network dynamics to the electromagnetic field in the extracellular space poses challenging problems; some of them have been addressed to some extent by and Bédard et al. (2004); Bédard and Destexhe (2009), and Bédard and Destexhe (2012).

In computer simulation studies, neural mass potentials, such as LFP and EEG are most realistically simulated by means of multicompartmental models (Protopapas et al., 1998; Sargsyan et al., 2001; Lindén et al., 2010; Lindén et al., 2011). Lindén et al., (2010) calculated the current dipole momentum of the DFP for single pyramidal and stellate cells, based on several hundreds compartments of the dendritic trees. Their results were in compliance with the standard dipole approximation of the electrostatic multipole expansion in the far-field (more than 1 mm remote from the dendritic trunk), but they found rather poor agreement with that approximation in the vicinity of the cell body. For comparison they also computed a “two-monopole” model of one synaptic current and its counterpart, the somatic return current, estimated from the current dipole momentum of the whole dendritic tree. This “two-monopole” model, which corresponds to an electrically equivalent single dipole model, obtained from the decomposition of the dendrite into two compartments, better approximates the true current dipole momentum in the vicinity of the pyramidal neuron. By superimposing the DFPs of pyramidal cells to the ensemble LFP, Lindén et al. (2011) found that LFP properties cannot be attributed to the far-field dipole approximation.

However, realistic multicompartmental models are computationally too expensive for large-scale neural network simulations. Therefore, various techniques have been proposed and employed to overcome computational complexity. These include networks of point models (i.e., devoid from any spatial representation), based on conductance models (Hodgkin and Huxley, 1952; Mazzoni et al., 2008), population density models (Omurtag et al., 2000), or firing rate models (Wilson and Cowan, 1972), which can be seen as a sub class of population density models, with uniform density distribution (Chizhov et al., 2007). In these kinds of models, mass potentials such as LFP or EEG are conventionally described as averaged membrane potential. A different class of models are neural mass models (Jansen and Rit, 1995; Wendling et al., 2000; David and Friston, 2003; Rodrigues et al., 2010), where mass potentials are estimated either through sums (or actually differences) of excitatory postsynaptic potentials (EPSP) (David and Friston, 2003) or of excitatory postsynaptic currents (EPSC) (Mazzoni et al., 2008).

In particular, the model of Mazzoni et al. (2008) which is based on Brunel and Wang (2003), recently led to a series of follow-up studies (Mazzoni et al., 2010, 2011) addressing the correlations between numerically simulated and experimentally measured LFP/EEG with spike rates by means of statistical modeling and information theoretic measures. In all of the above point models and their extension to population models, it is assumed that the extracellular space is iso-potential and the majority of studies thereby neglect the effect of extracellular resistance. That is, the extracellular space constitutes a different and isolated domain with no effect on neuronal dynamics.

In this article we extend the *ad hoc* model of Mazzoni et al. (2008) toward a biophysically better justified approach, taking the dipole character of extracellular currents and fields into account. Basically, our model corresponds to the “two-monopole,” or, equivalent dipole model of Lindén et al. (2010) which gave a good fit of the DFP close to the cell body of a cortical pyramidal neuron. However, we aim to keep the simplicity of the Mazzoni et al. (2008) model in terms of computational complexity, by endowing the extracellular space with resistance and by keeping point-like neuronal circuits. That is, in our case we do not quite consider point neurons, nor spatially extended models with detailed compartmental morphology, yet an intermediate level of description is achieved. To this end we propose a *reduced three-compartmental model* of a single pyramidal neuron (Destexhe, 2001; Wang et al., 2004; beim Graben, 2008), and derive an observation model for the dendritic dipole currents in the extracellular space and thereby for the DFP that contributes to the LFP of a neural population. Interestingly, our reduced three-compartmental model enables us to derive a leaky integrate-and-fire (LIF) mechanism [as for a point model (Mazzoni et al., 2008)], with additional observation equations for the DFP, which all together allows to study the relationship between spike rates and LFP. Our derivations also nicely map realistic electrotonic parameters to phenomenological parameters considered in Mazzoni et al. (2008).

## 2. Materials and methods

Mazzoni et al. (2008) consider three populations of neurons, namely excitatory cortical pyramidal cells (population 1), inhibitory cortical interneurons (population 2), and excitatory thalamic relay neurons (population 3), passing sensory input to the cortex that is simulated by a random (Erdős–Rényi) graph of *K* = 4000 pyramidal and *L* = 1000 interneurons with connection probability *P* = 0.2.

### 2.1. Theory

**Figure 2:**
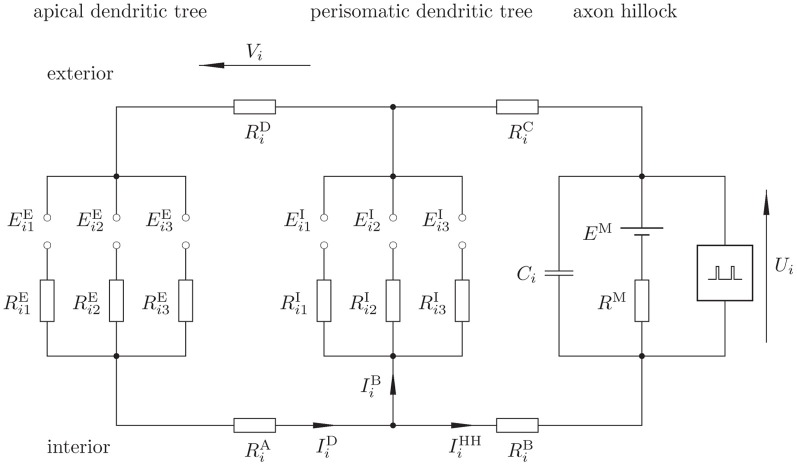
**Proposed electronic equivalent circuit for a pyramidal neuron (reduced three-compartmental model).** Note that the apical and basal dendrites are not true compartments since capacitors are not explicitly represented, rather, these are implicitly taken into account via EPSP and IPSP static functions, thus keeping computational complexity low.

We describe the *i*th cortical pyramidal neuron (Figure 1) from population 1 via the electronic equivalent (reduced) three-compartment model (Figure 2) (Destexhe, 2001; Wang et al., 2004; beim Graben, 2008), which is parsimonious to derive our observation model: one compartment for the apical dendritic tree, another one for soma and perisomatic basal dendritic tree (Lindén et al., 2010), and the third—actually a LIF unit—for the axon hillock where membrane potential is converted into spike trains by means of an integrate-and-fire mechanism.

Excitatory synapses are represented by the left-most branch, where EPSP at a synapse between a neuron *j* from population 1 or 3 and neuron *i* act as electromotoric forces *E*^E^_*ij*_. These potentials drive EPSC *I*^E^_*ij*_, essentially consisting of sodium ions, through the cell plasma with resistance *R*^E^_*ij*_ from the synapse toward the axon hillock.

The middle branch describes the inhibitory synapses between a neuron *k* from population 2 and neuron *i*. Here, inhibitory postsynaptic potentials (IPSP) *E*^I^_*ik*_ provide a shortcut between the excitatory branch and the trigger zone, where inhibitory postsynaptic currents (IPSC) *I*^I^_*ik*_ (essentially chloride ions) close the loop between the apical and perisomatic dendritic trees. The resistivity of the current paths along the cell plasma is given by *R*^I^_*ik*_.

The cell membrane at the axon hillock itself is represented by the branch at the right hand side. Here, a capacitor *C*_*i*_ reflects the temporary storage capacity of the membrane. The serial circuit consisting of a battery *E*^M^ and a resistor *R*^M^ denotes the Nernst resting potential and the leakage conductance of the membrane, respectively (Johnston and Wu, 1997). Finally, a spike generator (Hodgkin and Huxley, 1952; Mazzoni et al., 2008) (indicated by a “black box”) is regarded of having infinite input impedance. Both, EPSP and IPSP result from the interaction of postsynaptic receptor kinetics with dendritic low-pass filtering in compartments one and two, respectively (Destexhe et al., 1998; Lindén et al., 2010). Hence the required capacitances, omitted in Figure 2, are already taken into account by *E*^E^_*ij*_, E^I^_*ik*_. Therefore, we refer to our model as to a “reduced compartment model” here.

The three compartments are coupled through longitudinal resistors, *R*^A^_*i*_, *R*^B^_*i*_, *R*^C^_*i*_, and *R*^D^_*i*_ where *R*^A^_*i*_, *R*^B^_*i*_ denote the resistivity of the cell plasma and *R*^C^_*i*_, *R*^D^_*i*_ that of extracellular space (Holt and Koch, 1999).

Finally, the membrane voltage at the axon hillock *U*_*i*_ (the dynamical state variable) and the DFP *V*_*i*_, which measures the drop in electrical potential along the extracellular resistor *R*^D^_*i*_ are indicated. For the aim of calculation, the mesh currents *I*^D^_*i*_ (the dendritic current), *I*^B^_*i*_ (the basal current), and *I*^IF^_*i*_ (the integrate-and-fire current) are indicated.

The circuit in Figure 2 obeys the following equations:

(1) $$I_{i}^{\text{D}}=\sum_{j\;=\;1}^{p}I_{ij}^{\text{E}}$$

(2) $$I_{i}^{\text{B}}=\sum_{k\;=\;1}^{q}I_{ik}^{\text{I}}$$

(3) $$I_{i}^{\text{IF}}=I_{i}^{\text{D}}-I_{i}^{\text{B}}$$

(4) $$I_{i}^{\text{IF}}=C_{i}\frac{\text{d}U_{i}}{\text{d}t}+\frac{U_{i}-E^{\text{M}}}{R^{\text{M}}}$$

(5) $$E_{ij}^{\text{E}}=R_{ij}^{\text{E}}I_{ij}^{\text{E}}+\left(R_{i}^{\text{A}}+R_{i}^{\text{D}}\right)I_{i}^{\text{D}}+\left(R_{i}^{\text{B}}+R_{i}^{\text{C}}\right)I_{i}^{\text{IF}}+U_{i},1\leq j\leq p$$

(6) $$E_{ik}^{\text{I}}=R_{ik}^{\text{I}}I_{ik}^{\text{I}}+\left(R_{i}^{\text{B}}+R_{i}^{\text{C}}\right)I_{i}^{\text{IF}}+U_{i},1\leq k\leq q$$

(7) $$V_{i}=R_{i}^{\text{D}}I_{i}^{\text{D}}.$$

Here, *p* is the number of excitatory and *q* is the number of inhibitory synapses connected to neuron *i*.

The circuit described by Equations (1–7) shows that the neuron *i* is likely to fire when the excitatory synapses are activated. Then, the integrate-and-fire current *I*^IF^_*i*_ equals the dendritic current *I*^D^_*i*_. If, by contrast, also the inhibitory synapses are active, the dendritic current *I*^D^_*i*_ is shunted between the apical and perisomatic basal dendritic trees and only a portion could evoke spikes at the trigger zone (Equation 4). On the other hand, the large dendritic current *I*^D^_*i*_ flowing through the extracellular space of resistance *R*^D^_*i*_, gives rise to a large DFP *V*_*i*_.

In order to simplify the following derivations, we gauge the resting potential (Equation 4) to *E*^M^ = 0, yielding

(8) $$I_{i}^{\text{IF}}=C_{i}\frac{\text{d}U_{i}}{\text{d}t}+\frac{U_{i}}{R^{\text{M}}}.$$

From Equation (5) we obtain the individual EPSC's as

(9) $$I_{ij}^{\text{E}}=\frac{1}{R_{ij}^{\text{E}}}\left[E_{ij}^{\text{E}}-\left(R_{i}^{\text{A}}+R_{i}^{\text{D}}\right)I_{i}^{\text{D}}-\left(R_{i}^{\text{B}}+R_{i}^{\text{C}}\right)I_{i}^{\text{IF}}-U_{i}\right]\text{​}.$$

And accordingly, the individual IPSC's from Equation (6)

(10) $$I_{ik}^{\text{I}}=\frac{1}{R_{ik}^{\text{I}}}\left[E_{ik}^{\text{I}}-\left(R_{i}^{\text{B}}+R_{i}^{\text{C}}\right)I_{i}^{\text{IF}}-U_{i}\right]\text{​}.$$

Inserting Equation (9) into Equation (1) yields the excitatory dendritic current

(11) $$I_{i}^{\text{D}}=\sum_{j\;=\;1}^{p}\frac{1}{R_{ij}^{\text{E}}}E_{ij}^{\text{E}}-g_{i}^{\text{E}}\left[\left(R_{i}^{\text{A}}+R_{i}^{\text{D}}\right)I_{i}^{\text{D}}+\left(R_{i}^{\text{B}}+R_{i}^{\text{C}}\right)I_{i}^{\text{IF}}+U_{i}\right]\text{​},$$

where we have introduced the excitatory dendritic conductivity

(12) $$g_{i}^{\text{E}}=\sum_{j\;=\;1}^{p}\frac{1}{R_{ij}^{\text{E}}}.$$

Likewise we obtain the inhibitory dendritic currents from Equations (2) and (10) as

(13) $$I_{i}^{\text{B}}=\sum_{k\;=\;1}^{q}\frac{1}{R_{ik}^{\text{I}}}E_{ik}^{\text{I}}-g_{i}^{\text{I}}\left[\left(R_{i}^{\text{B}}+R_{i}^{\text{C}}\right)I_{i}^{\text{IF}}+U_{i}\right]\text{​},$$

with the inhibitory dendritic conductivity

(14) $$g_{i}^{\text{I}}=\sum_{k\;=\;1}^{q}\frac{1}{R_{ik}^{\text{I}}}.$$

With these results, we obtain an interface equation for an observation model as follows. Rearranging Equation (11) yields

(15) $$I_{i}^{\text{D}}\left[1+g_{i}^{\text{E}}\left(R_{i}^{\text{A}}+R_{i}^{\text{D}}\right)\right]=\sum_{j\;=\;1}^{p}\frac{1}{R_{ij}^{\text{E}}}E_{ij}^{\text{E}}-g_{i}^{\text{E}}\left[\left(R_{i}^{\text{B}}+R_{i}^{\text{C}}\right)I_{i}^{\text{IF}}+U_{i}\right]$$

Next, we eliminate *I*^IF^_*i*_ through Equation (8):

$$I_{i}^{\text{D}}\left[1+g_{i}^{\text{E}}\left(R_{i}^{\text{A}}+R_{i}^{\text{D}}\right)\right]=\sum_{j\;=\;1}^{p}\frac{1}{R_{ij}^{\text{E}}}E_{ij}^{\text{E}}-g_{i}^{\text{E}}\times \left[C_{i}\left(R_{i}^{\text{B}}+R_{i}^{\text{C}}\right)\frac{\text{d}U_{i}}{\text{d}t}+U_{i}\left(1+\frac{R_{i}^{\text{B}}+R_{i}^{\text{C}}}{R^{\text{M}}}\right)\right]\text{​}.$$

Division by 1 + *g*^E^_*i*_ (*R*^A^_*i*_ + *R*^D^_*i*_) gives the desired expression for the extracellular dendritic dipole current:

(16) $$I_{i}^{\text{D}}=\sum_{j\;=\;1}^{p}\alpha_{ij}E_{ij}^{\text{E}}-\beta_{i}\frac{\text{d}U_{i}}{\text{d}t}-\gamma_{i}U_{i},$$

with the following electrotonic parameters

(17) $$\alpha_{ij}=\frac{1}{R_{ij}^{\text{E}}\left[1+g_{i}^{\text{E}}\left(R_{i}^{\text{A}}+R_{i}^{\text{D}}\right)\right]}$$

(18) $$\beta_{i}=\frac{C_{i}g_{i}^{\text{E}}\left(R_{i}^{\text{B}}+R_{i}^{\text{C}}\right)}{1+g_{i}^{\text{E}}\left(R_{i}^{\text{A}}+R_{i}^{\text{D}}\right)}$$

(19) $$\gamma_{i}=\frac{g_{i}^{\text{E}}\left(R^{\text{M}}+R_{i}^{\text{B}}+R_{i}^{\text{C}}\right)}{R^{\text{M}}\left[1+g_{i}^{\text{E}}\left(R_{i}^{\text{A}}+R_{i}^{\text{D}}\right)\right]}.$$

In order to derive the evolution equation we consider the integrate-and-fire current *I*^IF^_*i*_ that is given through Equation (3). The individual EPSCs and IPSCs have already been obtained in Equations (9) and (10), respectively. Inserting Equation (13) into Equation (3) yields

$$I_{i}^{\text{IF}}\left[1-g_{i}^{\text{I}}\left(R_{i}^{\text{B}}+R_{i}^{\text{C}}\right)\right]-g_{i}^{\text{I}}U_{i}=I_{i}^{\text{D}}-\sum_{k\;=\;1}^{q}\frac{1}{R_{ik}^{\text{I}}}E_{ik}^{\text{I}}.$$

Next we insert our interface equation Equation (16) and also Equation (8):

$$\left[C_{i}\frac{\text{d}U_{i}}{\text{d}t}+\frac{U_{i}}{R^{\text{M}}}\right]\left[1-g_{i}^{\text{I}}\left(R_{i}^{\text{B}}+R_{i}^{\text{C}}\right)\right]-g_{i}^{\text{I}}U_{i}=\sum_{j\;=\;1}^{p}\alpha_{ij}E_{ij}^{\text{E}}-\beta_{i}\frac{\text{d}U_{i}}{\text{d}t}-\gamma_{i}U_{i}-\sum_{k\;=\;1}^{q}\frac{1}{R_{ik}^{\text{I}}}E_{ik}^{\text{I}}$$

and obtain after some rearrangements

$$\{C_{i}\left[1-g_{i}^{\text{I}}\left(R_{i}^{\text{B}}+R_{i}^{\text{C}}\right)\right]+\beta_{i}\}\frac{\text{d}U_{i}}{\text{d}t}+\frac{1-g_{i}^{\text{I}}\left(R_{i}^{\text{B}}+R_{i}^{\text{C}}+R^{\text{M}}\right)+R^{\text{M}}\gamma_{i}}{R^{\text{M}}}U_{i}=\sum_{j\;=\;1}^{p}\alpha_{ij}E_{ij}^{\text{E}}-\sum_{k\;=\;1}^{q}\frac{1}{R_{ik}^{\text{I}}}E_{ik}^{\text{I}}$$

and after multiplication with

$$r_{i}=\frac{R^{\text{M}}}{1-g_{i}^{\text{I}}\left(R_{i}^{\text{B}}+R_{i}^{\text{C}}+R^{\text{M}}\right)+R^{\text{M}}\gamma_{i}}$$

the dynamical law for the membrane potential at axon hillock:

(20) $$\tau_{i}\frac{\text{d}U_{i}}{\text{d}t}+U_{i}=\sum_{j\;=\;1}^{p}w_{ij}^{\text{E}}E_{ij}^{\text{E}}-\sum_{k\;=\;1}^{q}w_{ik}^{\text{I}}E_{ik}^{\text{I}},$$

where we have introduced the following parameters:

• *time constants*

(21) $$\tau_{i}=r_{i}\left\{C_{i}\left[1-g_{i}^{\text{I}}\left(R_{i}^{\text{B}}+R_{i}^{\text{C}}\right)\right]+\beta_{i}\right\}$$

• *excitatory synaptic weights*

(22) $$w_{ij}^{\text{E}}=r_{i}\alpha_{ij}$$

• *inhibitory synaptic weights*

(23) $$w_{ik}^{\text{I}}=\frac{r_{i}}{R_{ik}^{\text{I}}}.$$

Using the result Equation (20), we can also eliminate the temporal derivative in the interface equation Equation (16) through

(24) $$\frac{\text{d}U_{i}}{\text{d}t}=\frac{1}{\tau_{i}}\left[\sum_{j\;=\;1}^{p}w_{ij}^{\text{E}}E_{ij}^{\text{E}}-\sum_{k\;=\;1}^{q}w_{ik}^{\text{I}}E_{ik}^{\text{I}}-U_{i}\right]$$

which yields

$$I_{i}^{\text{D}}=\sum_{j\;=\;1}^{p}\left(\alpha_{ij}-\frac{\beta_{i}}{\tau_{i}}w_{ij}^{\text{E}}\right)E_{ij}^{\text{E}}+\sum_{k\;=\;1}^{q}\frac{\beta_{i}}{\tau_{i}}w_{ik}^{\text{I}}E_{ik}^{\text{I}}+\left(\frac{\beta_{i}}{\tau_{i}}-\gamma_{i}\right)\text{​}U_{i}.$$

And eventually, by virtue of Equation (7) after multiplication with *R*^D^_*i*_ the DFP

(25) $$V_{i}=\sum_{j\;=\;1}^{p}{\tilde{w}}_{ij}^{\text{E}}E_{ij}^{\text{E}}+\sum_{k\;=\;1}^{q}{\tilde{w}}_{ik}^{\text{I}}E_{ik}^{\text{I}}+\xi_{i}U_{i},$$

with parameters

(26) $${\tilde{w}}_{ij}^{\text{E}}=R_{i}^{\text{D}}w_{ij}^{\text{E}}\left(\frac{1}{r_{i}}-\frac{\beta_{i}}{\tau_{i}}\right)$$

(27) $${\tilde{w}}_{ik}^{\text{I}}=R_{i}^{\text{D}}w_{ik}^{\text{I}}\frac{\beta_{i}}{\tau_{i}}$$

(28) $$\xi_{i}=R_{i}^{\text{D}}\left(\frac{\beta_{i}}{\tau_{i}}-\gamma_{i}\right)\text{​}.$$

The change in sign of the inhibitory contribution from Equation (20) to Equation (25) has an obvious physical interpretation: In Equation (20), the change of membrane potential *U*_*i*_ and therefore the spike rate is enhanced by EPSPs but diminished by IPSPs. On the other hand, the dendritic shunting current *I*^D^_*i*_ in Equation (25) is large for both, large EPSPs and large IPSPs.

From Equation (20) we eventually obtain the neural network's dynamics by taking into account that postsynaptic potentials are obtained from presynaptic spike trains through temporal convolution with postsynaptic impulse response functions, i.e.,

(29) $$E_{ij}^{\text{E|I}}(t)=\int_{-\infty }^{t}s_{i}^{\text{E|I}}(t-t^{\prime })R_{j}(t^{\prime })\text{ d}t^{\prime }$$

where *s*^E|I^_*i*_(*t*) are excitatory and inhibitory synaptic impulse response functions, respectively, and *R*_*j*_ is the spike train

(30) $$R_{j}(t)=\sum_{t_{\nu }}\delta \left(t-t_{\nu }-\tau_{L}\right)$$

coming from presynaptic neuron *j*, when spikes were emitted at times *t*_ν_. The additional time constant τ_*L*_ is attributed to synaptic transmission delay (Mazzoni et al., 2008). These events are obtained by integrating Equation (20) with initial condition

(31) $$U_{i}\left(t_{\nu }\right)=E.$$

where *E* is some steady-state potential (Mazzoni et al., 2008). If at time *t* = *t*_ν_ the membrane reaches a threshold

(32) $$U_{i}(t)\geq \theta_{i}(t)$$

[with possibly a time-dependent activation threshold θ_*i*_(*t*)] from below $\frac{\text{d}U_{i}(t)}{\text{d}t}>0$ then an output spike δ(*t* − *t*_ν_) is generated, which is then followed by a potential resetting as follows

(33) $$U_{i}(t_{\nu +1})\leftarrow E.$$

Additionally, the integration of the dynamical law is restarted at time *t* = *t*_ν + 1_ + τ_*rp*_ after interrupting the dynamics for a refractory period τ_*rp*_.

Inserting Equation (29) into Equation (20) entails the evolution equation of the neural network

(34) $$\tau_{i}\frac{\text{d}U_{i}}{\text{d}t}+U_{i}=\sum_{j\;=\;1}^{p}w_{ij}^{\text{E}}s_{i}^{\text{E}}(t)*R_{j}(t)+\sum_{k\;=\;1}^{q}w_{ik}^{\text{I}}s_{i}^{\text{I}}(t)*R_{k}(t),$$

where the signs had been absorbed by the synaptic weights, such that *w*^E^_*ij*_ > 0 for excitatory synapses and *w*^I^_*ik*_ < 0 for inhibitory synapses, respectively.

Following Mazzoni et al. (2008) an individual postsynaptic current *I*^E|I^_*ij*_ at a synapse between neurons *i* and *j* obeys

(35) $$\tau_{d}^{\text{E|I}}\frac{\text{d}I_{ij}^{\text{E|I}}}{\text{d}t}+I_{ij}^{\text{E|I}}=x_{ij}^{\text{E|I}}$$

(36) $$\tau_{r}^{\text{E|I}}\frac{\text{d}x_{ij}^{\text{E|I}}}{\text{d}t}+x_{ij}^{\text{E|I}}=F_{ij}^{\text{E|I}},$$

where τ^E|I^_*d*_ are decay time constants and τ^E|I^_*r*_ are rise time constants of EPSC and IPSC, respectively. Auxiliary variables are denoted by *x*^E|I^_*ij*_, while *F*^E|I^_*ij*_ prescribes presynaptic forcing

(37) $$F_{ij}^{\text{E|I}}=\tau_{i}J_{ij}R_{j}(t)$$

with spike train Equation (30). Here, *J*_*ij*_ = *vw*^E|I^_*ij*_ denotes synaptic gain with *v* = 1 mV as voltage unit.

Note that Equation (37) is essentially a weighted sum of delta functions, such that a single spike can be assumed as particular forcing

(38) $$F=F_{0}\delta (t),$$

with some constant *F*_0_.

Derivating Equation (35) and eliminating *x*^E|I^_*ij*_ transforms Equations (35, 36) into a linear second-order differential equation with constant coefficients

(39) $$\tau_{d}^{\text{E|I}}\tau_{r}^{\text{E|I}}\frac{{\text{d}}^{2}I_{ij}^{\text{E|I}}}{\text{d}t^{2}}+\left(\tau_{d}^{\text{E|I}}+\tau_{r}^{\text{E|I}}\right)\frac{\text{d}I_{ij}^{\text{E|I}}}{\text{d}t}+I_{ij}^{\text{E|I}}=F_{ij}^{\text{E|I}}.$$

Equation (39) with the particular forcing Equation (38) is solved by a Green's function *s*^E|I^_*i*_(*t*) such that the general solution of Equation (39) is obtained as the temporal convolution

(40) $$I_{ij}^{\text{E|I}}(t)=\int_{-\infty }^{t}s_{i}^{\text{E|I}}(t-t^{\prime })F_{ij}^{\text{E|I}}(t)\text{ d}t^{\prime }.$$

For *t* ≠ 0, Equation (39) assumes its homogeneous form and is easily solved by means of the associated characteristic polynomial

(41) $$\tau_{d}^{\text{E|I}}\tau_{r}^{\text{E|I}}\lambda^{2}+\left(\tau_{d}^{\text{E|I}}+\tau_{r}^{\text{E|I}}\right)\lambda +1=0$$

with roots λ_1_ = −1/τ^E|I^_*d*_ and λ_2_ = −1/τ^E|I^_*r*_, entailing the Green's functions

(42) $$s_{i}^{\text{E|I}}(t)=\left(A^{\text{E|I}}{\text{e}}^{t/\tau_{r}^{\text{E|I}}}-B^{\text{E|I}}{\text{e}}^{t/\tau_{d}^{\text{E|I}}}\right)\Theta (t)$$

with the Heaviside step function Θ(*t*).

The constants *A*^E|I^, *B*^E|I^ > 0 are obtained from the initial conditions *s*^E|I^_*i*_(*t*) = 0, reflecting causality, and a suitable normalization

$$\int_{0}^{\infty }s_{i}^{\text{E|I}}(t)\text{ d}t=1.$$

The initial condition yields *A*^E|I^ = *B*^E|I^ ≡ *S*^E|I^, while the remaining constant

$$S^{\text{E|I}}=\frac{1}{\tau_{d}^{\text{E|I}}-\tau_{r}^{\text{E|I}}},$$

due to normalization. Therefore, the normalized Green's functions are those of Brunel and Wang (2003)

(43) $$s_{i}^{\text{E|I}}(t)=v\frac{\tau_{i}}{\tau_{d}^{\text{E|I}}-\tau_{r}^{\text{E|I}}}\left({\text{e}}^{t/\tau_{r}^{\text{E|I}}}-{\text{e}}^{t/\tau_{d}^{\text{E|I}}}\right)\Theta (t).$$

Now, we are able to compare our DFP *V*_*i*_ (Equation 25) with the estimate of Mazzoni et al. (2008) which is given (in our notation) as the sums of the moduli of excitatory and inhibitory synaptic currents, i.e.,

(44) $$V_{i}^{\text{MPLB}}=\sum_{j}|I_{ij}^{\text{E}}|+\sum_{k}|I_{ik}^{\text{I}}|$$

where “MPLB” refers to the authors Mazzoni et al. (2008).

From Equations (25) and (44), respectively, we compute two models of the LFP. First, by summing DFP across all pyramidal neurons (beim Graben and Kurths, 2008; Mazzoni et al., 2008), and, second by taking the DFP average (Nunez and Srinivasan, 2006), which yields

(45) $$L_{1}=\sum_{i}V_{i}^{\text{MPLB}}$$

(46) $$L_{2}=\frac{1}{K}\sum_{i}V_{i}^{\text{MPLB}}$$

(47) $$L_{3}=\sum_{i}V_{i}$$

(48) $$L_{4}=\frac{1}{K}\sum_{i}V_{i},$$

where *K* is number of pyramidal neurons.

### 2.2. Parameter estimation

Next, we relate the electrotonic parameters of our model to the phenomenological parameters of Mazzoni et al. (2008). To this end, we first report their synaptic efficacies in Table 1.

**Table 1:** **Parameters laid as in Mazzoni et al. (2008)**.

**Synaptic efficacies/mV**	**On interneurons**	**On pyramidal neurons**
GABA	2.7	1.7
Recurrent cortical AMPA	0.7	0.42
External thalamic AMPA	0.95	0.55

From these, we compute the synaptic weights through

(49) $$w_{ij}^{\text{E}}=J_{ij}^{\text{E}}/v=\{\begin{array}{cc} 0.42\text{ if} & j\;“\text{cortical}”\\ 0.55\text{ if} & j\;“\text{thalamic}” \end{array}$$

and

$$w_{ik}^{\text{I}}=J_{ik}^{\text{I}}/v=1.7$$

Next, we determine the factors *r*_*i*_ by virtue of Equation (23) through

$$r_{i}=\frac{w_{ik}^{\text{I}}}{{\bar{g}}_{\text{GABA}}}=\frac{1.7}{\text{1 nS}}=1.7\text{G}\Omega$$

using the inhibitory synaptic conductivity ${\bar{g}}_{\text{GABA}}=1\text{ nS}$, correspondingly, Equation (22) allows us to express α_*ij*_ in terms of the excitatory synaptic weights through

$$\alpha_{ij}=\frac{w_{ij}^{\text{E}}}{r_{i}}=\{\begin{array}{cc} 0.25\text{nS if} & j\;“\text{cortical}”\\ 0.32\text{nS if} & j\;“\text{thalamic}” \end{array}$$

From α_*ij*_ we can determine the total excitatory synaptic conductivities *g*^E^_*i*_ according to Equation (17) through

(50) $$\begin{array}{c} \alpha_{ij}=\frac{1}{R_{ij}^{\text{E}}\left[1+g_{i}^{\text{E}}\left(R_{i}^{\text{A}}+R_{i}^{\text{D}}\right)\right]}\;\\ g_{i}^{\text{E}}\left[1-\left(R_{i}^{\text{A}}+R_{i}^{\text{D}}\right)\sum_{j=1}^{p}\alpha_{ij}\right]=\sum_{j=1}^{p}\alpha_{ij}\\ g_{i}^{\text{E}}=\frac{\sum_{j=1}^{p}\alpha_{ij}}{1-\left(R_{i}^{\text{A}}+R_{i}^{\text{D}}\right)\sum_{j=1}^{p}\alpha_{ij}} \end{array}$$

and hence

(51) $$R_{ij}^{\text{E}}=\frac{1}{\alpha_{ij}\left[1+g_{i}^{\text{E}}\left(R_{i}^{\text{A}}+R_{i}^{\text{D}}\right)\right]}$$

Inserting next Equation (18) into Equation (21) yields

(52) $$\begin{array}{l} \tau_{i}=r_{i}C_{i}\frac{1+g_{i}^{\text{E}}\left(R_{i}^{\text{A}}\text{​}+\text{​}R_{i}^{\text{D}}\right)\text{​}+\text{​}\left(R_{i}^{\text{B}}+R_{i}^{\text{C}}\right)\left\{g_{i}^{\text{E}}-g_{i}^{\text{I}}\left[1+g_{i}^{\text{E}}\left(R_{i}^{\text{A}}+R_{i}^{\text{D}}\right)\right]\right\}}{1+g_{i}^{\text{E}}\left(R_{i}^{\text{A}}+R_{i}^{\text{D}}\right)}. \end{array}$$

Equation (52) could constraint the choice of the membrane capacitance *C*_*i*_ by choosing τ_*i*_ = 20ms (Mazzoni et al., 2008).

In order to also determine the DFP parameters Equations (26–28), we finally compute the ratios

$$\frac{\beta_{i}}{\tau_{i}}=\frac{g_{i}^{\text{E}}\left(R_{i}^{\text{B}}+R_{i}^{\text{C}}\right)}{r_{i}\left\{1\text{​}+g_{i}^{\text{E}}\left(R_{i}^{\text{A}}+R_{i}^{\text{D}}\right)\text{​}+\text{​}\left(R_{i}^{\text{B}}\text{​}+R_{i}^{\text{C}}\right)\left\{g_{i}^{\text{E}}\text{​}-\text{​}g_{i}^{\text{I}}\left[1+g_{i}^{\text{E}}\left(R_{i}^{\text{A}}+R_{i}^{\text{D}}\right)\right]\right\}\right\}}.$$

The remaining electrotonic parameters *R*^M^_*i*_, *R*^A^_*i*_, *R*^B^_*i*_, *R*^C^_*i*_, and *R*^D^_*i*_ are estimated from cell geometries as follows. The resistance *R* of a volume conductor is proportional to its length ℓ and reciprocally proportional to its cross-section *A*, i.e.,

(53) $$R=\rho \frac{\ell }{A}$$

where ρ is the (specific) resistivity of the medium. Table 2 shows the resistivities of the three kinds of interest which then allows to evaluate the various volume conductor resistances according to Equation (53).

**Table 2:** **Resistivities of cell membrane, cell plasma and extracellular space**.

**Medium**	***ρ*/Ω cm**
Cell membrane (at axon hillock)	5 × 10^7^
Cell plasma (cytoplasm)	200
Extracellular space	333

*Parameters from Rall (1977); Mainen et al. (1995); Kole and Stuart (2012), and Gold et al. (2007). Note that the resistivity of the cell membrane has to be related to the constant membrane thickness (≈10 nm)*.

We consider a total dendritic length of 2ℓ = 20 μ m and a dendritic radius of *a* = 7 μ m, that are generally subjected to variation. Equally, parameters that were allowed to vary are the length and radius of the axon hillock, yet herein we consider a length of 2ℓ = 20 μ m and radius of *a* = 0.5 μ m (Mainen et al., 1995; Destexhe, 2001; Kole and Stuart, 2012). To evaluate the intracellular (*R*_*A*_, *R*_*B*_) and extracellular (*R*_*D*_, *R*_*C*_) resistances, respectively, according to Equation (53), we consider a simple implementation where the length ℓ is half of the dendritic length (i.e., basal and apical length are symmetrical, but this can be broken). However, the cross sectional area for the cytoplasm is simply *A* = π*a*^2^. Finally, the area of the axon hillock is simply the surface area of a cylinder.

In order to also determine the cross-section of extracellular space between dendritic trunks we make the following approximations. We assume that dendritic trunks are parallel aligned cylinders of radius *a* and length ℓ that are hexagonally dense packed. Then the centers of three adjacent trunks form an equilateral triangle with side length 2a and hence area $2\sqrt{3}a^{2}$. The enclosed space is then given by the difference of the triangle area and the area of three sixth circle sectors, therefore

$$A_{\text{space}}=2\sqrt{3}a^{2}-\frac{3}{6}\pi a^{2}=\left(2\sqrt{3}-\frac{1}{2}\pi \right)a^{2}.$$

Hence, the cross-section of extracellular space surrounding one trunk is

(54) $$A=6A_{\text{space}}=\left(12\sqrt{3}-3\pi \right)a^{2}.$$

### 2.3. Simulations

Subsequently, we implement an identical network to the one considered by Mazzoni et al. (2008) with *Brian Simulator*, that is a Python-based environment (Goodman and Brette, 2009). However, the derivations from the previous section enables the possibility of setting a dipole observable that measures the local DFP on each pyramidal neurons, given by Equation (25). This allows then to define a mesoscopic LFP observable, which can be equated either as averaged DFP or simply given as the sum of DFP, given by Equations (45–48). Primarily, we compare our LFP measure *L*_4_, proposed as the average of DFP, with the Mazzoni et al. LFP *L*_1_ which is defined as the sum of absolute values of GABA and AMPA currents (Equation 44). Additionally, we also compare all possible measures, namely, mean membrane potential $\frac{1}{K}\sum_{i}U_{i}$, Mazzoni et al. LFP *L*_1_, average of Mazzoni et al. DFP *L*_2_, sum of DFP *L*_3_, and the average of DFP *L*_4_.

For completeness, we briefly summarize the description of the network [we refer the reader to Mazzoni et al. (2008) for details]. The network models a cortical tissue with LIF neurons, composed of 1000 inhibitory interneurons and 4000 pyramidal neurons, which are described by the evolution Equation (34). The threshold crossings given by Equation (32) is considered static with θ_*i*_ = 18 mV and the reset potential *E* = 11 mV. The refractory period for excitatory neurons is τ_*rp*_ = 2 ms while for inhibitory neurons it is τ_*rp*_ = 1 ms. The network connectivity is random and sparse with a 0.2 probability of directed connection between any pair of neurons. The evolution of synaptic currents, fast GABA (inhibitory) and AMPA (excitatory) are described via the second order evolution Equations (35, 36), which are activated by incoming presynaptic spikes represented by Equation (30). The latency of the postsynaptic currents is set to τ_*L*_=1 ms and the rise and decay times are given by Table 3.

**Table 3:** **Synaptic rise (τ_*r*_) and decay times (τ_*d*_)**.

**Synaptic times**	**τ_*r*_/ms**	**τ_*d*_/ms**
GABA	0.25	5
AMPA on interneurons	0.2	1
AMPA on pyramidal neurons	0.4	2

Parameters laid as in Mazzoni et al. (2008)

Moreover, synaptic efficacies, *J*^E|I^_*ij*_, for simulation were presented in Table 1. Note that Relation (49) then allows to determine the synaptic weights. Additionally, all neurons receive external thalamic excitatory inputs, that is, via AMPA-type synapses, which are activated by random Poisson spike trains, with a time varying rate that is identical for all neurons. Specifically, the thalamic inputs are the only source of noise, which attempts to account for both cortical heterogeneity and spontaneous activity. This is achieved by modeling a two level noise, where the first level is an Ornstein–Uhlenbeck process superimposed with a constant signal and the second level is a time varying inhomogeneous Poisson process. Thus, we have the following time varying rate, λ(*t*), that feeds into inhomogeneous Poisson process:

(55) $$\tau_{n}\frac{\text{d}n(t)}{\text{d}t}=-n(t)+\sigma_{n}\sqrt{\frac{2}{\tau_{n}}}\eta (t)$$

(56) $$\lambda (t)={\left[c_{0}+n(t)\right]}_{+}$$

where η(*t*) represents Gaussian white noise, *c*_0_ represents a constant signal (but equally could be periodic or other), and the operation [·] is the threshold-linear function, [x]_+_ = x if *x* > 0, [x]_+_ = 0 otherwise, which circumvents negative rates. The constant signal *c*_0_ can range between 1.2 and 2.6 spikes/ms. The parameters of the Ornstein–Uhlenbeck process are τ_*n*_ = 16 ms and the standard deviation σ_*n*_=0.4 spikes/ms.

For complete exposition, we note that from an implementation viewpoint (within the Brian simulator), a copy of the postsynaptic impulse response function (Equation 29) has to be evaluated to calculate the DFP (Equation 25) with weights ${\tilde{w}}_{ij}^{\text{E|I}}$. This implies evaluating the second order process (Equations 35, 36) with a different forcing term. Specifically, starting from *I*^E|I^_*ij*_(*t*) ≡ *w*^E|I^_*ij*_ *E*^E|I^_*ij*_(*t*) = *s*^E|I^_*i*_(*t*) * *F*^E|I^_*ij*_ and pre-multiplying both sides with ${\tilde{w}}_{ij}^{\text{E|I}}$ and subsequently re-arranging we obtain the desired forcing term ${\tilde{F}}_{ij}^{\text{E|I}}={\tilde{w}}_{ij}^{\text{E|I}}F_{ij}^{\text{E|I}}/w_{ij}^{\text{E|I}}$. Note further that by expanding the term *F*^E|I^_*ij*_ with Equation (37) and using Relation (49) we finally obtain ${\tilde{F}}_{ij}^{\text{E|I}}={\tilde{w}}_{ij}^{\text{E|I}}\tau_{i}vR_{j}(t)$.

## 3. Results

**Figure 3:**
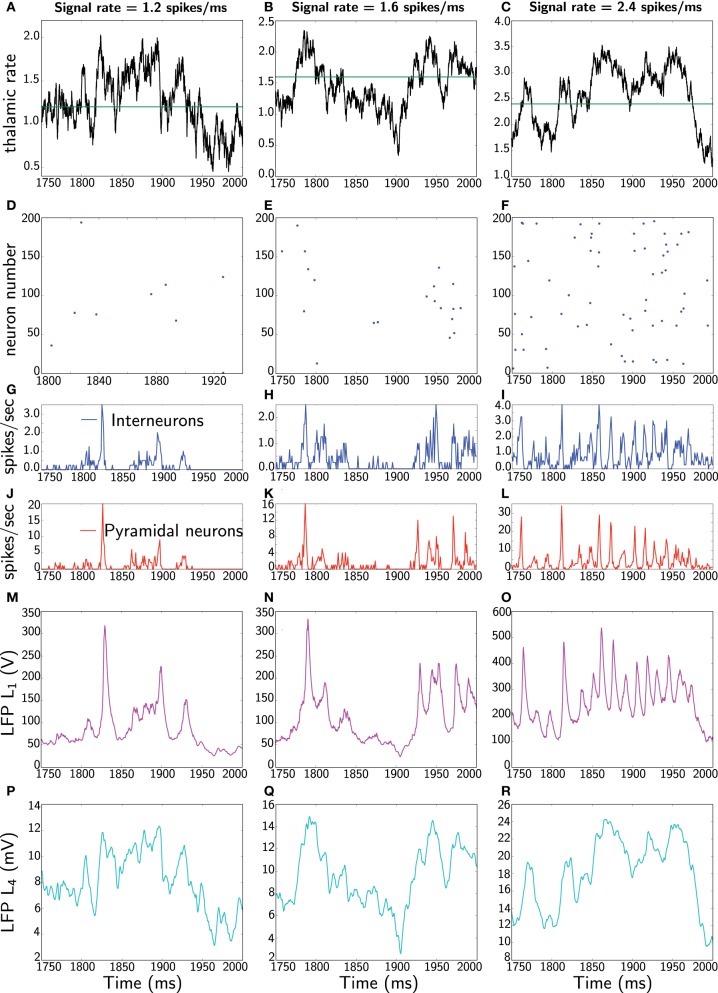
**Dynamics of the network and LFP comparisons: the three columns represent different runs of the network for three different rates, 1.2, 1.6, and 2.4 spikes/ms.** In each column, all panels show the same 250 ms (extracted from 2 s simulations). The first panels **(A–C)** represent thalamic inputs with the different rates. The second panels **(D–F)** corresponds to a raster plot of the activity of 200 pyramidal neurons. The third panels **(G–I)** depict average instantaneous firing rate (computed on a 1 ms bin) of interneurons (blue) and fourth panels **(J–L)** correspond to average instantaneous firing rate of pyramidal neurons. The fifth panels **(M–O)** show the Mazzoni et al. LFP *L*_1_ from Equation (45). Finally, the last panels **(P–R)** depict our proposed LFP measure *L*_4_, which is the average of dendritic field potential (DFP) (Equation 48).

Following Mazzoni et al. (2008), the network simulations are run for 2 s with three different noise levels, specifically, receiving a constant signal with three different rates 1.2, 1.6, and 2.4 spikes/ms as depicted in Figure 3. Note that these input rates do not mean that a single neuron fires at these high rates. Rather, it can be obtained from multiple neurons that jointly fire with slower, yet desynchronized, rates converging at the same postsynaptic cell. The Poisson process ensures that this is well represented.

The focus is to compare our proposed measure *L*_4_, defined as mean of the DFP (Equation 48), with the Mazzoni et al. LFP *L*_1_ from Equation (45). In Figure 3 one sees two main striking differences between the two measures, namely in frequency and in amplitude. Specifically, *L*_1_ responds instantaneously to the spiking network activity by means of high frequency oscillations. Moreover, *L*_1_ also exhibits a large amplitude. In contrast, our mean DFP *L*_4_ measures comparably to experimental LFP, that is, in the order of millivolts, and although it responds to population activity, it has a relatively smoother response. Actually one can realize that our LFP estimate represents low-pass filtered thalamic input.

The physiological relevance of this is not yet clear in our work. However, recent work (Poulet et al., 2012) shows that desynchronized cortical state during active behavior is driven by a centrally generated increase in thalamic action potential firing (i.e., thalamic firing controls cortical states). Thus, it seems that cortical synchronous activity is suppressed when thalamic input increases, thereby suggesting that cortical desynchronized states to be related to sensory processing. This work further quantifies these observations by applying Fast Fourier Transform (FFT) to cortical EEG and subsequently comparing with thalamic firing rate by means of Pearson correlation coefficient. Unfortunately they do not quantify the amount of thalamic oscillations contained within the cortical EEG.

**Figure 4:**
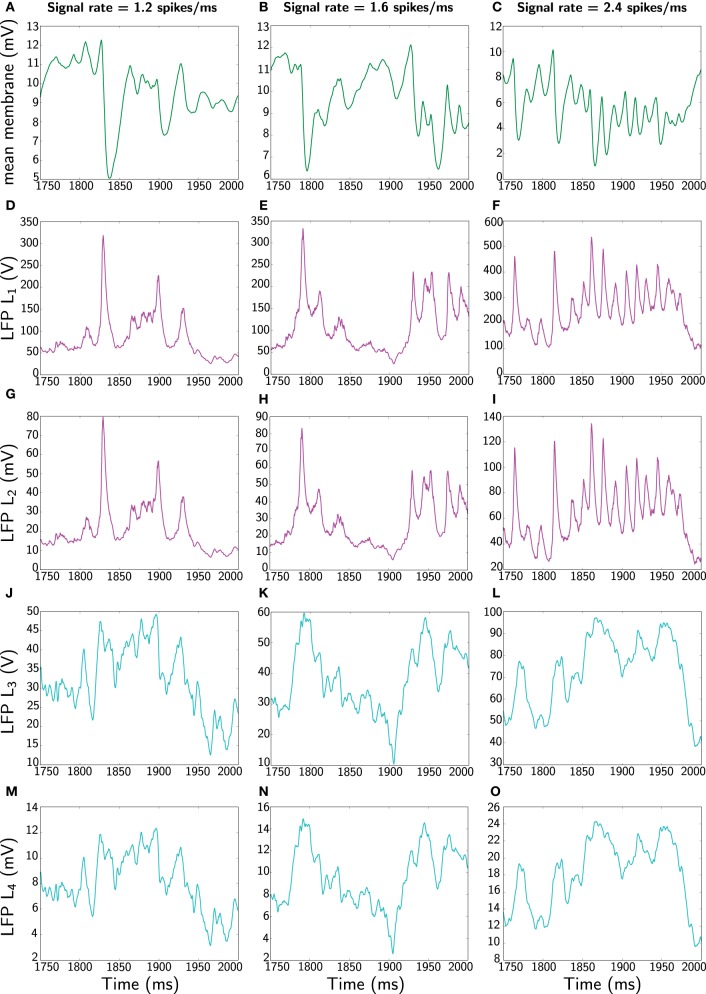
**Comparison of different LFP measures when the network receives constant signal with three different rates (1.2, 1.6, and 2.4 spikes/ms).** Again, only 250 ms is represented (extracted from 2 s simulation). The first panels **(A–C)** corresponding to the different rates shows the most widespread LFP measure used in the literature, namely average membrane potential $\frac{1}{K}\sum_{i}U_{i}$. The second panels **(D–F)** shows the Mazzoni et al. LFP *L*_1_ from Equation (45). The third panels **(G–I)** displays the average of the Mazzoni et al. DFP *L*_2_ (Equation 46). Similarly, the fourth panels **(J–L)** shows the total, *L*_3_, (Equation 47) and the last panels **(M–O)** depicts the averaged, *L*_4_, (Equation 48) LFP measure. Note the different amplitude scales between measures.

Yet, to keep a comparable comparison between measures, we also compute the average of the Mazzoni et al. DFP *L*_2_ (Equation 48) and additionally the mean membrane potential (the standard considered in the neuroscientific literature). These are shown in Figure 4.

Clearly, in terms of time profile, the summed and averaged observables are similar within the same class of LFP measures. However, in all cases the Mazzoni et al. LFP *L*_1_ exhibits a significantly larger order of magnitude, which diverges substantially from experimental LFP amplitudes, typically varying between 0.5 and 2 mV (Lakatos et al., 2005; Niedermeyer, 2005). In contrast, although the mean DFP is not contained within the interval from 0.5 to 2 mV it arguably performs better. However, we do concede further work is required. Some gains in improving the different LFP measures can be achieved by applying for example a weighted average, which would mimic the distance of an electrode to a particular neuron by means of a lead field kernel (Nunez and Srinivasan, 2006). For example, a convolution of either *L*_1_ or *L*_2_ with a Gaussian kernel (representing the distance to a neuron), would yield a measure that captures better the LFP or better the DFP of the nearest neurons. However, further work will be required to properly quantify the gain when space is taken into account.

**Figure 5:**
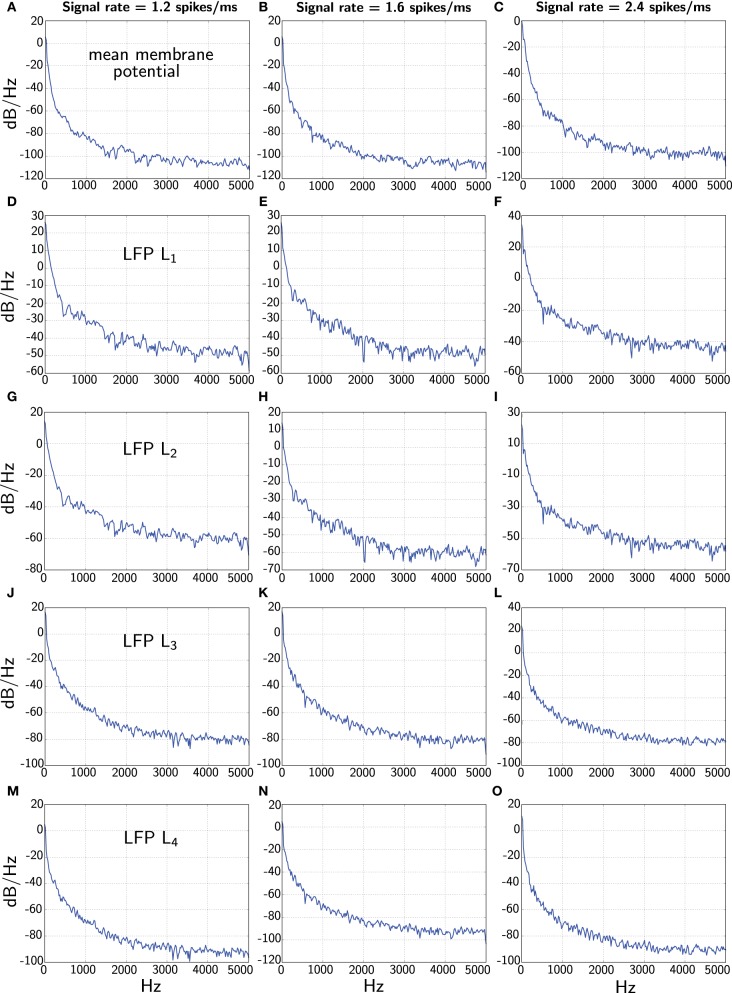
**Comparison of power spectra of the various LFP measures when the network receives constant signal with three different rates (1.2, 1.6, and 2.4 spikes/ms).** The first panels **(A–C)** corresponding to the different rates shows the power spectrum of the average membrane potential $\frac{1}{K}\sum_{i}U_{i}$. The second panels **(D–F)** and third panels **(G–I)** show power spectra of the total and average of *L*_1_ and *L*_2_ corresponding to Mazzoni et al. (2008), respectively. The fourth panels **(J–L)** and the last panels **(M–O)** display power spectra of the *L*_3_ and *L*_4_ measures from our model, respectively. Note we show the full spectrum up to 5 kHz only for convenience due to the fine sample rate.

In Figure 5 we finally contrast the power spectra of the different LFP measures.

One interesting feature is that the power spectrum of the Mazzoni et al. LFP measures decays much more slowly that the average membrane potential for higher frequencies. This observation is true for both, *L*_1_ and *L*_2_. In contrast, our LFP measures *L*_3_ and *L*_4_ fare better, and in particular, *L*_4_ decays at an approximately similar rate as the average membrane potential.

## 4. Discussion

In this article we derived a model for cortical dipole fields, such as DFP/LFP from biophysical principles. To that aim we decomposed a cortical pyramidal cell, the putative generator of those potentials, into three compartments: the apical dendritic tree as the place of mainly excitatory (AMPA) synapses, the soma and the perisomatic dendritic tree as the place of mainly inhibitory (GABA) synapses, and the axon hillock as the place of wave-to-spike conversion by means of an integrate-and-fire mechanism. From Kirchhoff's laws governing an electronic equivalent circuit of our model, we were then able to derive the evolution equation for neural network activity (Equation 34) and, in addition, an observation equation (25) for the dendritic dipole potential contributing to the LFP of a cortical population.

In order to compare our approach with another model discussed in the recent literature (Mazzoni et al., 2008, 2010, 2011) we aligned the parameters of our model with the model of Mazzoni et al. (2008) who approximated DFP as the sum of moduli of excitatory and inhibitory synaptic currents (Equation 44). From both approaches, we computed four different LFP estimates: *L*_1_, the sum of Mazzoni et al. DFP, *L*_2_, the population average of Mazzoni et al. DFP, *L*_3_ the sum of our dipole DFP, and *L*_4_ the population average of our dipole DFP (Equations 45–48).

Our results indicate two main effects between our dipole LFP measures and those of Mazzoni et al. Firstly, the measures based on Mazzoni et al. (2008) systematically overestimate LFP amplitude by almost one order of magnitude. One reason for that could be attributed to the direct conversion of synaptic current into voltage without taking extracellular conductivity into account, as properly done in our approach. Yet, another, even more crucial reason is disclosed by our equivalent circuit (Figure 2). In our approach there is just *one* extracellular current *I*^D^ flowing from the perisomatic to the apical dendritic tree. In the model of Mazzoni et al. (2008), however, two synaptic currents that might be of the same order of magnitude are superimposed to the DFP. Secondly, the measures based on Mazzoni et al. (2008) also systematically overestimate LFP frequencies. This could probably be attributed partly to spurious higher harmonics introduced by computing absolute values. Moreover, taking the power spectrum shows that the Mazzoni et al. (2008) measure decays much more slowly than the average membrane potential, which is at variance with experimental data.

However, at the current stage, both models, that of Mazzoni et al. (2008) and our own, agree with respect to the polarity of DFP and LFP. The measures based on Mazzoni et al. (2008) have positive polarity simply due to the moduli. On the other hand, also the direction of current dipoles in our model is constrained by the construction of the equivalent circuit (Figure 2) where current sources are situated at the perisomatic and current sinks are situated at apical dendritic tree. Taking this polarity as positive also entails positive DFP and LFP that could only change in strength. However, it is well known from brain anatomy that pyramidal cells appear in at least two layers, III and VI, of neocortex. This is reflected in experiments when an electrode traverses different layers by LFP polarity reversals, and, of course, by the fact that LFP and EEG oscillate between positive and negative polarity. Adapting our model to this situation could be straightforwardly accomplished in the framework of neural field theory by fully representing space and simulating layered neural fields (Amari, 1977; Jirsa and Haken, 1996; beim Graben, 2008). By contrast such a generalization is impossible at all with the model of Mazzoni et al. (2008) due to the presence of absolute values.

On theses grounds we have good indication that our measure is an improvement to the Mazzoni et al. LFP measures, and, quite importantly, it is biophysically better motivated than the *ad hoc* model of Mazzoni et al. (2008). However, much considerable effort is still required to underpin all the relevant LFP mechanisms and to better represent experimental LFP/EEG dynamics.

Finally, our work provides a new framework where DFPs and the relationship between firing rates and local fields can be explored without the extreme demand on computational complexity involved in multicompartmental modeling (Protopapas et al., 1998; Sargsyan et al., 2001; Lindén et al., 2010; Lindén et al., 2011) by adopting reduced compartment circuits. For example, we envisage to extend our recent work which maps firing rate model (derived from LIF models) to population density models (Chizhov et al., 2007), but now incorporating our observational DFP model. In addition, our framework is analytically amenable and thus can be applied to any linear differential equation, for instance, GIF (Gif-sur-Yvette Integrate Fire) models, which are improvements to the LIF models and compute more accurately spike activations (Rudolph-Lilith et al., 2012). Also resonant membranes (mediated by Ca^2+^ and a Ca^2+^-activated K^+^ ionic currents) that describe sub-threshold oscillations and which can be easily expressed by linear equations (Mauro et al., 1970) can be incorporated in our derivations. We note, however, that our framework can be applied to non-linear equations, with Hodgkin and Huxley (1952) type activation, but it will fall short from explicit and analytical observation equations.

### Conflict of interest statement

The authors declare that the research was conducted in the absence of any commercial or financial relationships that could be construed as a potential conflict of interest.

## References

[B1] Amari S.-I. (1977). Dynamics of pattern formation in lateral-inhibition type neural fields. Biol. Cybern. 27, 77–87 911931 10.1007/BF00337259

[B2] Bédard C. Destexhe A. (2009). Macroscopic models of local field potentials and the apparent 1/f noise in brain activity. Biophys. J. 96, 2589–2603 10.1016/j.bpj.2008.12.3951 19348744 PMC2711281

[B3] Bédard C. Destexhe A. (2012). Modeling local field potentials and their interaction with the extracellular medium, in Handbook of Neural Activity Measurement, eds Brette R. Destexhe A. (Cambridge, MA: Cambridge University Press), 136–191

[B4] Bédard C. Kröger H. and Destexhe A. (2004). Modeling extracellular field potentials and the frequency-filtering properties of extracellular space. Biophys. J. 86, 1829–1842 10.1016/S0006-3495(04)74250-2 14990509 PMC1304017

[B5] beim Graben P. (2008). Foundations of neurophysics, in Lectures in Supercomputational Neuroscience: Dynamics in Complex Brain Networks, Springer Complexity Series, eds Graben P. b. Zhou C. Thiel M. Kurths J. (Berlin: Springer), 3–48

[B6] beim Graben P. Kurths J. (2008). Simulating global properties of electroencephalograms with minimal random neural networks. Neurocomputing 71, 999–1007 4672572

[B7] Berger H. (1929). Über das Elektroenkephalogramm des Menschen. Archiv für Psychiatrie 87, 527–570

[B8] Brunel N. Wang X.-J. (2003). What determines the frequency of fast network oscillations with irregular neural discharges? I. synaptic dynamics and excitation-inhibition balance. J. Neurophysiol. 90, 415–430 10.1152/jn.01095.2002 12611969

[B9] Chizhov A. V. Rodriguesb S. Terryb J. R. (2007). A comparative analysis of a firing-rate model and conductance-based neural population model. Phys. Lett. A 369, 31–36

[B10] Creutzfeldt O. D. Watanabe S. and Lux H. D. (1966a). Relations between EEG phenomena and potentials of single cortical cells. I. evoked responses after thalamic and epicortical stimulation. Electroencephalogr. Clin. Neurophysiol. 20, 1–18 4161317 10.1016/0013-4694(66)90136-2

[B11] Creutzfeldt O. D. Watanabe S. and Lux H. D. (1966b). Relations between EEG phenomena and potentials of single cortical cells. II. spontaneous and convulsoid activity. Electroencephalogr. Clin. Neurophysiol. 20, 19–37 4161316 10.1016/0013-4694(66)90137-4

[B12] David O. Friston K. J. (2003). A neural mass model for MEG/EEG: coupling and neuronal dynamics. Neuroimage 20, 1743–1755 10.1016/j.neuroimage.2003.07.015 14642484

[B13] Destexhe A. (2001). Simplified models of neocortical pyramidal cells preserving somatodendritic voltage attenuation. Neurocomputing 38–40, 167–173

[B14] Destexhe A. Mainen F. Sejnowski T. J. (1998). Kinetic models of synaptic transmission, in Methods in Neuronal Modelling. From Ions to Networks, eds Koch C. Segev I. (Cambridge, MA: MIT Press), 1–25

[B15] Gold C. Henze D. Koch C. (2007). Using extracellular action potential recordings to constrain compartmental models. J. Comput. Neurosci. 23, 39–58 10.1007/s10827-006-0018-2 17273940

[B16] Goodman D. Brette R. (2009). The Brian simulator. Front. Neurosci. 3, 192–197: 10.3389/neuro.01.026.2009 20011141 PMC2751620

[B17] Hodgkin A. L. Huxley A. F. (1952). A quantitative description of membrane current and its application to conduction and excitation in nerve. J. Physiol. 117, 500–544 12991237 10.1113/jphysiol.1952.sp004764 PMC1392413

[B18] Holt G. R. Koch C. (1999). Electrical interactions via the extracellular potential near cell bodies. J. Comput. Neurosci. 6, 169–184 10333161 10.1023/a:1008832702585

[B19] Jansen B. H. Rit V. G. (1995). Electroencephalogram and visual evoked potential generation in a mathematical model of coupled cortical columns. Biol. Cybern. 73 357–366. 7578475 10.1007/BF00199471

[B20] Jirsa V. K. Haken H. (1996). Field theory of electromagnetic brain activity. Phys. Rev. Lett. 77, 960–963 10.1103/PhysRevLett.77.960 10062950

[B21] Johnston D. Wu S. M.-S. (1997). Foundations of Cellular Neurophysiology. Cambridge, MA: MIT Press

[B22] Koch C. Segev I. (eds.). (1998). Methods in Neuronal Modelling. From Ions to Networks, 2nd Edn., Computational Neuroscience. Cambridge, MA: MIT Press

[B23] Kole M. Stuart G. (2012). Signal processing in the axon initial segment. Neuron 73, 235–247 10.1016/j.neuron.2012.01.007 22284179

[B24] Lakatos P. Shah A. Knuth K. Ulbert I. Karmos G. Schroeder C. (2005). An oscillatory hierarchy controlling neuronal excitability and stimulus processing in the auditory cortex. J. Neurophysiol. 94, 1904–1911 10.1152/jn.00263.2005 15901760

[B25] Lindén H. Pettersen K. Einevoll G. (2010). Intrinsic dendritic filtering gives low-pass power spectra of local field potentials. J. Comput. Neurosci. 29, 423–444 10.1007/s10827-010-0245-4 20502952

[B26] Lindén H. Tetzlaff T. Potjans T. C. Pettersen K. H. Grün S. Diesmann M. (2011). Modeling the spatial reach of the LFP. Neuron 72, 859–872 10.1016/j.neuron.2011.11.006 22153380

[B27] Mainen Z. Joerges J. Huguenard J. Sejnowski T. (1995). A model of spike initiation in neocortical pyramidal neurons. Neuron 15, 1427–1439 10.1016/0896-6273(95)90020-9 8845165

[B28] Mauro A. Conti F. Dodge F. Schor R. (1970). Subthreshold behavior and phenomenological impedance of the squid giant axon. J. Gen. Physiol. 55, 497–532 5435782 10.1085/jgp.55.4.497 PMC2203007

[B29] Mazzoni A. Brunel N. Cavallari S. Logothetis N. K. Panzeri S. (2011). Cortical dynamics during naturalistic sensory stimulations: experiments and models. J. Physiol. 105, 2–15 10.1016/j.jphysparis.2011.07.014 21907800

[B30] Mazzoni A. Panzeri S. Logothetis N. K. Brunel N. (2008). Encoding of naturalistic stimuli by local field potential spectra in networks of excitatory and inhibitory neurons. PLoS Comput. Biol. 4:e1000239 10.1371/journal.pcbi.1000239 19079571 PMC2585056

[B31] Mazzoni A. Whittingstall K. Brunel N. Logothetis N. K. Panzeri S. (2010). Understanding the relationships between spike rate and delta/gamma frequency bands of LFPs and EEGs using a local cortical network model. Neuroimage 52, 956–972 10.1016/j.neuroimage.2009.12.040 20026218

[B32] Niedermeyer E. (2005). The normal EEG of the waking adult, in Electroencephalography: Basic Principles, Clinical Applications, and Related Fields, 5th Edn, eds Niedermeyer E. Silva F. L. D. (Philadelphia, PA: Lippincott Williams & Wilkins), 167–192

[B33] Nunez P. L. Srinivasan R. (2006). Electric Fields of the Brain: The Neurophysics of EEG. 2nd Edn New York, NY: Oxford University Press

[B34] Omurtag A. Knight B. Sirovich L. (2000). On the simulation of large populations of neurons. J. Comput. Neurosci. 8, 51–63 10798499 10.1023/a:1008964915724

[B35] Poulet J. F. Fernandez L. M. Crochet S. Petersen C. C. (2012). Thalamic control of cortical states. Nat. Neurosci. 15, 370–372 10.1038/nn.3035 22267163

[B36] Protopapas A. Vanier M. Bower J. M. (1998). Simulating large networks of neurons, in Methods in Neuronal Modelling. From Ions to Networks, eds Koch C. Segev I. (Cambridge, MA: MIT Press), 461–498

[B37] Rall W. (1977). Core conductor theory and cable properties of neurons, in Handbook of Physiology – The Nervous System, Cellular Biology of Neurons, Vol. 1, ed Kandel E. R. (Bethesda, MD: American Physiological Society), 39–97

[B38] Rodrigues S. Chizhov A. Marten F. Terry J. (2010). Mappings between a macroscopic neural-mass model and a reduced conductance-based model. Biol. Cybern. 102, 361–371 10.1007/s00422-010-0372-z 20306202

[B39] Rudolph-Lilith M. Dubois M. Destexhe A. (2012). Analytical integrate-and-fire neuron models with conductance-based dynamics and realistic postsynaptic potential time course for event-driven simulation strategies. Neural Comput. 34, 1426–1461 10.1162/NECO_a_00278 22364504

[B40] Sargsyan A. R. Papatheodoropoulos C. Kostopoulos G. K. (2001). Modeling of evoked field potentials in hippocampal CA1 area describes their dependence on NMDA and GABA receptors. J. Neurosci. Methods 104, 143–153 10.1016/S0165-0270(00)00334-4 11164240

[B41] Schomer D. L. Lopes da Silva F. H. (eds.). (2011). Niedermayer's Electroencephalography. Basic Principles, Clinical Applications, and Related Fields. 6th Edn Philadelphia, PA: Lippincott Williams & Wilkins

[B42] Spruston N. (2008). Pyramidal neurons: dendritic structure and synaptic integration. Nat. Rev. Neurosci. 9, 206–221 10.1038/nrn2286 18270515

[B43] Wang X.-J. Tegnér J. Constantinidis C. Goldman-Rakic P. S. (2004). Division of labor among distinct subtypes of inhibitory neurons in a cortical microcircuit of working memory. Proc. Natl. Acad. Sci. U.S.A. 101, 1368–1373 10.1073/pnas.0305337101 14742867 PMC337059

[B44] Wendling F. Bellanger J. J. Bartolomei F. Chauvel P. (2000). Relevance of nonlinear lumped-parameter models in the analysis of depth-EEG epileptic signals. Biol. Cybern. 83, 367–378 11039701 10.1007/s004220000160

[B45] Wilson H. R. Cowan J. D. (1972). Excitatory and inhibitory interactions in localized populations of model neurons. Biophys. J. 12, 1–24 10.1016/S0006-3495(72)86068-5 4332108 PMC1484078

